# Association Between Upper Extremity Function and Independence in Activities of Daily Living in Individuals with Motor-Incomplete Tetraplegia: An Exploratory Cross-Sectional Study

**DOI:** 10.3390/jfmk11010119

**Published:** 2026-03-16

**Authors:** Eleanna Strongylou, Dimitra Karadimitri, Maria Moutzouri, Magdalini Stamou, Christina-Anastasia Rapidi, Yannis Dionyssiotis, Eleni Moumtzi-Nakka, Vasiliki Sakellari

**Affiliations:** 1 Physiotherapy Department, School of Health and Care Sciences, University of West Attica, Agiou Spidonos 28, Egaleo, 12243 Athens, Greece; mscphys23015@uniwa.gr (E.S.); dkaradimitri@uniwa.gr (D.K.); moutzouri@uniwa.gr (M.M.); mstamou@uniwa.gr (M.S.); 2 Athens General Hospital Georgios Gennimatas, Leoforos Mesogion 154, 11527 Athens, Greece; rapidicha@hotmail.com; 3 2nd Physical Medicine & Rehabilitation Department, National Rehabilitation Center of Greece ΕΚA, Spyrou Theologou 1 & Filis Avenue, Ilion, 13122 Athens, Greece; yannis_dionyssiotis@hotmail.com; 4 Military Hospital for Special Diseases 414, Taxiarchou Veliou 414, 152 36 Penteli 6-8, Penteli, 15236 Athens, Greece; eleni.nakka@gmail.com

**Keywords:** exploratory pilot study, SCI rehabilitation, PRTEE, SCIM III, self-reported outcome measures, functional independence, cervical spinal cord injury, convenience sampling

## Abstract

**Background:** Spinal cord injury (SCI) is a leading cause of chronic disability. Loss of upper extremity (U.E.) function is central to limitations, in mobility, postural control, transfers, and self-care. The aim of this exploratory pilot study was to investigate whether self-reported UE function is associated with independence in activities of daily living (ADLs) in people with motor-incomplete tetraplegia. **Methods:** Eleven (*n* = 11) individuals with motor-incomplete tetraplegia (AIS C–D; neurological levels C4–T1; injury duration ≥ 1 year), recruited through convenience sampling from five specialist rehabilitation centres, participated in an exploratory cross-sectional pilot study designed to generate hypotheses rather than test them. U.E. function was assessed using the Patient-Rated Tennis Elbow Evaluation (PRTEE) questionnaire, selected for its ability to capture pain and task-related functional difficulty in the elbow, wrist, and hand; its application in this neurological population is considered exploratory. Independence in ADLs was evaluated using the Spinal Cord Independence Measure III (SCIM III). Given the small sample, all analyses were primarily descriptive and along with bivariate associations (Spearman correlations). Regression findings are reported strictly for exploratory purposes. **Results:** The median age was 50 years (interquartile range [IQR] 43–55). A strong negative correlation was observed between PRTEE total score and SCIM III (r_s_ = −0.76). In an exploratory univariate analysis, each 1-point increase in PRTEE total score was associated with a 1.3-point lower SCIM III score (β = −1.3, 95% CI −2.34 to −0.26, *p* = 0.02). Age also showed a positive association (β = 1.31, 95% CI 0.04 to 2.58, *p* = 0.05) with SCIM III; however, this finding is highly likely to reflect a statistical artefact of the small and unrepresentative sample. Multivariable regression was not conducted as a primary analysis due to insufficient statistical power. All findings should be treated as strictly exploratory and hypothesis-generating. **Conclusions:** Self-reported U.E. function appears to be associated with ADL independence in motor-incomplete tetraplegia. U.E. capacity may contribute to functional tasks requiring postural stability and mobility-related activities, but no predictive inferences can be made from this underpowered, convenience sample. Future studies with larger cohorts and performance-based measures are needed to confirm these preliminary observations and clarify the role of U.E. function in rehabilitation planning.

## 1. Introduction

SCI remains a major cause of long-term disability, affecting an estimated 15 million people worldwide and accounting for more than 4.5 million years lived with disability [1]. Epidemiological data indicate that incomplete tetraplegia is the most common category of SCI, representing 47.2% of all cases [2]. Individuals with cervical lesions typically exhibit weakness, spasticity, and motor and sensory deficits of the upper and lower extremities, trunk, and pelvic organs that compromise motor control, self-care, postural stability, transfer ability, and community participation [3].

Among the various impairments associated with SCI, the loss of U.E. and hand function has been identified as a key determinant of quality of life in individuals with tetraplegia [4,5]. Even modest improvements in elbow and hand control can substantially enhance self-care, transfers, wheelchair propulsion, making restoration of U.E. function a primary rehabilitation goal [6,7]. Effective U.E. function involves grasping and releasing objects, appropriately orienting the limb during object approach, recruiting suitable muscles, and applying adequate force. Bilateral coordination is also essential, as most ADLs require bimanual performance [8]. Beyond task execution, adequate UE strength and coordination contribute directly to postural control by enabling weight shifting, protective reactions, and pressure relief maneuver capacities that are fundamental for safe mobility and fall prevention in individuals with tetraplegia [9]. More specifically, U.E. loading during transfers, wheelchair propulsion, and weight-bearing activities generates co-contraction demands on trunk musculature, thereby contributing to segmental stabilization of the thoracic and lumbar spine [10,11,12]. In addition, adequate trunk strength is required to provide a stable proximal base for major shoulder muscles—such as the pectoralis major and latissimus dorsi, which originate from the thoracic spine—to generate effective force during wheelchair propulsion and seated postural control [13]. These interactions may directly mediate independence in mobility-related activities. In individuals with motor-incomplete tetraplegia, preserved yet weakened U.E. motor function may still elicit anticipatory postural adjustments, including feedforward mechanisms that pre-activate trunk stabilizers prior to voluntary limb movements, thereby coupling arm function with core stability [14,15]. Impaired force transmission through the kinetic chain from hand to trunk can therefore compromise not only task execution but also the postural foundation required for coordinated movement [7,9]. This biomechanical interdependence provides a theoretical basis for expecting associations between self-reported U.E. functional difficulty and multidimensional independence outcomes, including domains beyond self-care such as respiration management and mobility.

Several studies have examined the relationship between U.E. function and independence in individuals with cervical or thoracic SCI [16,17,18]. Rudhe et al. reported a strong correlation (r = 0.8, *p* < 0.1) between the self-care subscale of SCIM III and the GRASSP hand ability tests [16]. Similarly, a study assessing the reliability of the Capabilities of Upper Extremity Test (CUE-T) found a strong correlation (r = 0.7, κ > 0.6) between CUE-T and the SCIM III self-care subscale [17]. More recently, a 2023 study demonstrated a moderate correlation (r = 0.6, *p* < 0.01) between SCIM III self-care scores and wrist movement parameters—including duration, smoothness, and joint angle—during a drinking task, captured via motion analysis [18]. Collectively, these findings suggest that U.E. capacity may influence multiple domains of functional independence, including tasks requiring trunk control and coordinated movement during transfers and mobility.

Despite this emerging evidence, the relationship between self-reported U.E. functional capacity and ADL independence in individuals with chronic motor-incomplete tetraplegia remains insufficiently understood. Most prior investigations have relied predominantly on performance-based assessments, whereas validated patient-reported outcome measures (PROMs) adapted for cervical SCI populations remain scarce, despite their clinical practicality. Moreover, the measurement equivalence of upper limb instruments originally developed for musculoskeletal conditions has not been systematically evaluated in individuals with motor-incomplete tetraplegia. Consequently, simple clinic-ready indicators of functional independence are lacking.

Given the methodological challenges inherent in studying relatively small and heterogeneous clinical populations, exploratory investigations may provide valuable preliminary insights and help generate hypotheses for future confirmatory research. This gap is particularly evident in the limited examination of validated PROMs assessing U.E. function in relation to functional independence outcomes measured by instruments such as the SCIM III. While performance-based tools—including GRASSP, CUE-T, and kinematic analyses—have been examined against SCIM III scores [16,17,18], PROMs capturing the patient’s own perception of pain and task-related difficulty in U.E. activities have received substantially less attention in this population. This distinction is clinically relevant, as PROMs can be administered in routine outpatient settings without specialised equipment, offering potential value as low-burden screening tools for functional status.

Within an exploratory framework, determining whether self-reported U.E. difficulty correlates with ADL independence may provide hypothesis-generating insight into the potential clinical utility of such measures and inform patient-centered rehabilitation planning. Accordingly, the present study should be interpreted as an exploratory cross-sectional pilot investigation designed to generate, rather than test, hypotheses in a specialized and difficult-to-recruit clinical population (*N* = 11, convenience sampling). The small convenience sample is a recognized limitation that restricts statistical power and generalizability; these constraints are explicitly acknowledged throughout the manuscript.

Among UE assessment tools available in Greek, the Patient-Rated Tennis Elbow Evaluation (PRTEE) has demonstrated high internal consistency and construct validity in musculoskeletal populations [19,20]. Although originally developed for lateral elbow tendinopathy, the PRTEE captures pain and task-related difficulty across activities involving the elbow, wrist, and hand functions frequently compromised in tetraplegia. Its use in neurological populations remains limited; therefore, its application in the present study should be considered exploratory. Importantly, the instrument assesses both symptom burden and perceived functional ability, potentially offering a broader perspective on activity limitations [21]. Nevertheless, differences in underlying pathology between tendinopathy and SCI necessitate cautious interpretation, as construct validity for this population has not been firmly established.

Likewise, the SCIM III, also validated in Greek, has shown high internal consistency and construct validity [22,23]. Beyond self-care, the SCIM III evaluates respiration, sphincter management, and mobility domains, thereby capturing functional abilities closely related to posture, balance, and transfer performance. This makes it particularly suitable for examining how UE function may relate to multidimensional independence following cervical SCI.

Given the limited sample sizes typically accessible in specialized SCI cohorts, the development of predictive models may be statistically unstable. Therefore, the present study was designed as an exploratory cross-sectional pilot study designed to generate hypotheses rather than test them, emphasizing associations and effect estimates rather than causal inference or prediction.

The present study aimed (i) to examine the association between PRTEE and SCIM III scores (total and subscales) in individuals with chronic motor-incomplete tetraplegia (AIS C/D, C4–T1), and (ii) to explore the potential contribution of demographic and clinical factors to functional independence. We hypothesized that poorer self-reported UE function would be associated with lower ADL independence; however, any observed relationships were intended to be interpreted as hypothesis-generating rather than predictive.

## 2. Materials and Methods

### 2.1. Study Design

This exploratory cross-sectional pilot study, designed to generate hypotheses rather than test them, was approved by the Ethics and Deontology Committee of the University of West Attica (Decision No. 77266—2 September 2025. All participants provided written informed consent in accordance with the Declaration of Helsinki prior to participation.

Potential participants were initially contacted via telephone, during which the study objectives, procedures, and expectations were explained. Upon confirmation of eligibility, participants received the Information and Consent Form along with paper versions of the PRTEE, the SCIM III, and an anthropometric characteristics questionnaire via prepaid registered mail. Completion of all questionnaires required ap-proximately one hour. Participants could contact the primary investigator by telephone or online for clarifications. Completed questionnaires were returned using prepaid postage. For participants with limited upper extremity function, caregivers assisted in completing the forms, or the research team conducted a face-to-face interview to support accurate data capture. To minimise reporting bias and maintain standardised data collection across administration modes, a structured protocol was implemented. When caregiver assistance was necessary, caregivers were instructed to read questionnaire items verbatim, without providing interpretation, clarification, or rephrasing. Interview-based administration by the research team adhered strictly to the original questionnaire wording, with no paraphrasing or elaboration permitted. No proxy responses were accepted under any circumstances; all answers were required to reflect the participant’s own reported experience and judgment. Participants were specifically instructed that their responses should reflect their own perceptions, not those of caregivers or family members. No systematic differences in response patterns were observed between self-completed and assisted administration modes; however, formal subgroup comparison was not conducted due to the small sample size, which represents a limitation of the present study.

### 2.2. Setting

Recruitment was conducted through collaborating institutions, including the National Rehabilitation Center of Greece (EKA), General Hospital of Athens “Georgios Gennimatas”, General Hospital of Attica “KAT”, General Hospital of Chios “Skylitseio”, and the Military Hospital for Special Diseases “414”, Athens. Recruitment flyers were distributed between December 2024 and June 2025.

### 2.3. Participants

Eligible participants were adults (>18 years) (i) diagnosed with traumatic or non-traumatic SCI at neuro-logical levels C4–T1, (ii) classified as grade C or D on the ASIA Impairment Scale, (iii) with injury duration ≥1 year, and (iv) demonstrating limited independence in ADLs (SCIM III total score <100) [24].

Exclusion criteria included (i) psychological, neurological, or musculoskeletal comorbidities likely to substantially affect function, (ii) metabolic or cardiovascular disorders or orthopedic surgery resulting in reduced autonomy, and (iii) spasticity ≥3 on the Modified Ashworth Scale [25].

Given the specialized clinical population and recruitment constraints, a convenience sampling strategy was employed. Specifically, participants were recruited through convenience sampling from five collaborating specialist rehabilitation institutions (see Setting, Section 2.2). Potential participants identified through institutional records were contacted by telephone; those confirmed eligible were enrolled consecutively until data collection closed. No random selection or stratification was applied. The final sample comprised 11 individuals (all males; median age 50 years, IQR 43–55), all classified as AIS C (*n* = 7) or AIS D (*n* = 4), with neurological levels ranging from C4 to T1 and injury duration of at least one year. The convenience sampling approach was chosen because the target population (chronic motor-incomplete tetraplegia, AIS C/D, residing in the community) is inherently small and geographically dispersed; probabilistic sampling was not feasible within the scope of this pilot study. The sampling method and resulting sample size constitute a recognised limitation of the study and are discussed accordingly in Section 4.6.

### 2.4. Measures

#### 2.4.1. Anthropometric Characteristics

A 10-item questionnaire collected information on participants’ demographic characteristics, injury-related characteristics, use and type of walking aids, and weekly physical activity levels.

#### 2.4.2. Physical Activity Level

Physical activity was categorized into 4 levels, based on weekly sports and general exercise hours. Specifically, Level 1 (0 h, sedentary), Level 2 (1–3 h, moderately active), Level 3 (3–6 h, active), and Level 4 (>6 h, very active/athlete) [26].

#### 2.4.3. Upper Extremity Function

U.E. function was assessed using the PRTEE [21], a self-reported instrument evaluating perceived pain and task difficulty during manual activities.

The questionnaire consists of 15 items across two subscales: pain (5 items) and function (10 items). Each item is scored from 0 to 10, with higher scores indicating worse pain or difficulty. The total score ranges from 0 to 100.

For the purposes of this study, participants were instructed to consider both upper extremities when rating pain and function, reflecting the bilateral demands of postural control, wheelchair use, and transfer activities.

One item originally assessing difficulty at work was modified to evaluate difficulty using a mobile phone and computer due to the high proportion of retired participants. This pragmatic adaptation was intended to preserve item relevance; however, it may affect measurement equivalence and should be interpreted cautiously.

When items were not applicable (e.g., carrying a grocery bag), scores were imputed using the mean of the corresponding subscale in line with general scoring recommendations for handling missing PRTEE responses. Nevertheless, such imputation may introduce measurement bias and represent a methodological limitation. Internal consistency of the PRTEE in the present sample was assessed using Cronbach’s α. The total scale demonstrated acceptable internal consistency (α = 0.87), with the pain subscale (α = 0.85) and function subscale (α = 0.88) showing similarly adequate reliability. Caregiver assistance was limited to reading items verbatim without interpretation; no proxy responses were permitted, and all answers reflected the participant’s own report.

#### 2.4.4. Independence in Activities of Daily Living

Independence in ADLs was evaluated using the self-reported SCIM III [27], a validated instrument assessing self-care, respiration and sphincter management, and mobility. Scores range from 0 to 100, with higher values indicating greater independence.

Importantly, the mobility domain includes bed mobility and transfers, which rely heavily on U.E. strength and coordination for postural stabilization and safe movement. This supports its relevance for examining functional relationships aligned with posture and balance.

### 2.5. Outcome Measures

The primary outcome was independence in ADLs (SCIM III total score). U.E. function (PRTEE total and subscale scores) was treated as the principal explanatory variable within an exploratory analytic framework rather than as a predictor.

SCIM III subscales and participant characteristics (e.g., age, ASIA grade, activity level, walking aid) were examined as additional explanatory factors.

### 2.6. Bias

To reduce selection bias, recruitment occurred across multiple hospitals. Reporting bias was mitigated through the use of standardized instruments and availability of researcher support.

However, reliance on self-reported questionnaires introduces the possibility of recall and perception bias, which should be considered when interpreting the findings.

### 2.7. Statistical Analysis

The required sample size was estimated using a standardized multiple linear regression approach, consistent with the methodology of a previous study examining correlations among quantitative variables in individuals with spinal cord injury [28]. The significance level (α) was set at 0.05, statistical power (1 − β) at 0.90, and the anticipated effect size at 0.30. Based on these parameters a minimum of 29 participants with SCI was calculated to support adequately powered analyses [29].

An a priori sample size calculation therefore indicated that 29 participants would be required for multivariable regression. However, the final sample (*N* = 11) did not meet this threshold; accordingly, the statistical analyses were interpreted within an exploratory, hypothesis-generating framework consistent with an exploratory cross-sectional pilot study designed to generate hypotheses rather than test them.

Continuous variables, which did not follow a normal distribution, are presented as median and interquartile range (IQR, Q1–Q3). Categorical variables are reported as absolute frequencies and percentages (%).

Spearman’s rank correlation coefficient (r_s_) was used to examine associations between variables. No correction for multiple comparisons was applied; all correlation analyses are strictly exploratory and hypothesis-generating. Confidence intervals (95% CI) for correlation coefficients are reported alongside *p*-values. Correlation coefficients are presented without categorical interpretation.

Given that the final sample (*N* = 11) did not meet the a priori threshold of 29 participants required for multivariable regression, formal regression modeling was not conducted as a primary analysis. In accordance with the commonly cited rule of thumb of approximately 10–15 observations per predictor variable (Babyak, 2004) [30], only one explanatory variable (PRTEE total score) was entered into a univariate linear regression model with total SCIM III score as the dependent variable. Univariate regression results are reported for exploratory purposes only and should not be interpreted as predictive models. Results are strictly hypothesis-generating. The implications of this underpowering are explicitly addressed in the Discussion (Section 4.6, Limitations). In brief: (i) the study is substantially underpowered relative to the a priori sample size calculation (*N* = 11 vs. required *N* = 29), increasing the risk of Type II error and unstable effect estimates; (ii) regression coefficients from the exploratory univariate regression should be interpreted as descriptive summaries of within-sample associations only, not as stable population-level estimates; (iii) all effect size estimates are exploratory and hypothesis-generating in nature and should not be generalised beyond similar clinical contexts without replication in adequately powered samples.

All statistical analyses were performed using IBM SPSS Statistics, version 30.0.0 (IBM Corp., Armonk, NY, USA). The significance level was set at 5% for all tests.

No missing questionnaires were observed; however, item-level imputation was performed for non-applicable PRTEE responses. A total of 4 out of 11 participants (36%) required imputation for at least one item (mean of 1.5 items imputed per affected participant), primarily for items involving activities not applicable to their daily routine (e.g., carrying a grocery bag).

## 3. Results

### 3.1. Participant Characteristics

A total of 11 individuals with incomplete tetraplegia completed all study assessments. Participants were all male, with a median age of 50 years (IQR = 43–55, range 24–70). Median body weight and height were 82 kg (IQR = 70–98) and 180 cm (IQR = 172–186), respectively. Most participants used a powered wheelchair for mobility (*n* = 5), and the majority engaged in some form of weekly physical activity (*n* = 9) (Table 1).

Given the exploratory nature of the study and the limited sample size, participant characteristics are presented descriptively. Inferential *p*-values have been removed from descriptive tables, as no formal comparison groups were defined; tables present descriptive statistics only.

Table 2 presents the injury characteristics of the sample. Participants sustained SCI at neurological levels C4-T1, with ASIA grade C (*n* = 7) or D (*n* = 4). Median time since injury was 12 years (IQR = 7–16). Etiologies included traffic accidents (45%), myelopathy (36%), and sports-related injuries (18%).

### 3.2. Assessment Scores

Participants’ self-reported upper extremity function and pain, assessed via the PRTEE, are presented in Table 3. Median total PRTEE score was 46 (IQR = 41–59.5), with subscale medians of 19 for pain (IQR = 10–24) and 25 for function (IQR = 20–40). For items not applicable to participants’ daily routines—such as carrying a grocery bag—scores were imputed using the mean of the respective subscale in accordance with general scoring recommendations [21]. Because imputation may introduce measurement bias, these scores should be interpreted cautiously. Additionally, as the majority of participants (10 of 11) were retired, the item originally assessing difficulty at work was modified to evaluate difficulty using a mobile phone and computer. This pragmatic modification may affect measurement equivalence and should be considered when interpreting the findings. Participants’ SCIM III scores are summarized in Table 4. Median total SCIM III score was 54 (IQR = 25–74), with subscale medians of 10 (IQR = 1–16) for self-care, 26 (IQR = 21–35) for respiration and sphincter management, and 17 (IQR = 3–27) for mobility.

### 3.3. Associations Between Independence in Activities of Daily Living, Upper Extremity Function, Demographic Characteristics, and Injury-Related Factors

Total SCIM III score showed a negative correlation with the total PRTEE score (r_s_ = −0.76, 95% CI: −0.94 to −0.28, *p* = 0.01), the PRTEE function subscale (r_s_ = −0.93, 95% CI: −0.98 to −0.68, *p* < 0.01) and a positive correlation with the ASIA scale (r_s_ = 0.84, 95% CI: 0.47 to 0.96, *p* < 0.01). It also showed associations with participants’ age (r_s_ = 0.71, 95% CI: 0.17 to 0.92, *p* = 0.02) and the walking aid type used (r_s_ = 0.65, 95% CI: 0.07 to 0.90, *p* = 0.03). In contrast, only small correlations were observed between the SCIM III total score and the PRTEE pain subscale, body weight, height, years living with SCI, cause of injury, and activity level. The SCIM III self-care subscale showed good correlation with the total PRTEE score (rs = −0.69, *p* = 0.02) and age (r_s_ = 0.65, *p* = 0.03), and very good with the PRTEE function subscale (rs = −0.94, *p* < 0.01) and ASIA score (r_s_ = 0.86, *p* < 0.01). Similarly, the respiration and sphincter management subscale of SCIM III showed good correlation with age (r_s_ = 0.65, *p* = 0.03) and ASIA score (r_s_ = 0.6, *p* = 0.05), and a negative correlation with both total PRTEE score (r_s_ = −0.95, 95% CI: −0.99 to −0.74, *p* < 0.01) and PRTEE function subscale score (r_s_ = −0.83, 95% CI: −0.96 to −0.43, *p* < 0.01). Finally, the mobility subscale of SCIM III showed a negative correlation with the total PRTEE score (r_s_ = −0.74, 95% CI: −0.93 to −0.24, *p* = 0.01), participants’ age (r_s_ = 0.692, *p* = 0.018), and the walking aid type (r_s_ = 0.66, *p* = 0.03), and with the PRTEE function subscale score (r_s_ = −0.91, 95% CI: −0.98 to −0.62, *p* < 0.01) and the ASIA score (r_s_ = 0.85, *p* < 0.01). Overall, none of the SCIM III subscales showed a significant correlation with the PRTEE pain subscale. Note that no correction for multiple comparisons was applied; all findings should be interpreted as exploratory. Detailed correlations between SCIM III subscale scores, demographic characteristics, injury-related characteristics, and PRTEE subscale scores are presented in Table 5.

Given that the strongest correlations were observed between the PRTEE function subscale and both SCIM III total score and the self-care subscale score, the analysis further examined the relationship between the individual items of the PRTEE specific activity subscale and these SCIM III scores. As presented in Table 6, these correlations ranged from moderate to large in magnitude. Specifically, SCIM III self-care subscale score showed negative correlations with nearly all specific activity items, except for “open a jar” and “turn a doorknob/key”. The strongest association was observed for the item “wring out a washcloth/wet towel” (r_s_ = −0.86, 95% CI: −0.97 to −0.49, *p* < 0.01). Similarly, total SCIM III score showed negative correlations with the items “pull up pants” (r_s_ = −0.86, *p* < 0.01) and “wring out a washcloth/wet towel” (r_s_ = −0.83, *p* < 0.01). It should be noted that some PRTEE items (e.g., “pull up pants”) conceptually overlap with SCIM III tasks (e.g., dressing), which may have inflated correlation estimates. A sensitivity analysis excluding these overlapping items yielded slightly attenuated but directionally consistent correlations (PRTEE function subscale vs. SCIM III total: r_s_ = −0.88, *p* < 0.01), suggesting that the association is not entirely attributable to item overlap. These findings should nonetheless be interpreted with caution given the small sample.

### 3.4. Exploratory Regression Analysis

Considering the limited sample size (*N* = 11) and the increased risk of overfitting in multivariable models, we restricted the analysis to a univariate linear regression, with the PRTEE total score entered as the explanatory variable and the total SCIM III score as the dependent variable. This decision was guided by methodological recommendations (rule of thumb) indicating that approximately 10–15 observations per predictor are required to obtain more stable coefficient estimates [20]. Regression coefficients should be interpreted with caution as they are derived from an underpowered sample and are likely to be unstable.

In Table 7 the univariate regression coefficient for PRTEE total score is reported for exploratory purposes only. The univariate linear regression indicated that total PRTEE score showed an association with the total SCIM III score (β = −1.3, 95% CI: −2.34 to −0.26, *p* = 0.02). Age was also examined in a separate univariate model and showed an association with total SCIM III score (β = 1.31, 95% CI: 0.04 to 2.58, *p* = 0.05). These associations should be interpreted as exploratory observations only; given the small sample size, these coefficients are likely unstable and do not support predictive inference. Multivariable regression was not conducted as a primary analysis due to insufficient statistical power.

The univariate model should be considered a descriptive representation of associations within this specific dataset only and is not suitable for generalisation or clinical prediction.

**Table 7:** Univariate regression coefficients. Participants’ SCIM III Score (*N* = 11).

Predictor	Unadjusted β (95% CI)	*p*-Value
PRTEE Total Score	−1.3 (−2.34 to −0.26)	0.02
Age	1.31 (0.04 to 2.58)	0.05
Weight	0.37 (−0.46 to 1.2)	0.34

According to the univariate model, the following regression equation was derived:

(1) SCIM III = 112.29 − 1.3 × PRTEE

This equation describes the direction of the observed association within the present dataset only. Higher PRTEE scores were associated with lower SCIM III scores. Given the small sample size, this coefficient should be interpreted cautiously and viewed as descriptive rather than predictive.

The multivariable model including PRTEE total score, age, and weight (R^2^ = 0.56) is reported below for transparency, but is not interpreted as a primary finding due to the high risk of overfitting with *N* = 11:

(2) SCIM III = 5.91 − 0.81 × PRTEE + 1.04 × Age

In this model, PRTEE scores demonstrated a negative association with SCIM III, while age showed a positive association. However, the regression estimates are likely unstable due to the limited sample and should be regarded as exploratory mathematical representations of this cohort rather than tools suitable for individual estimation or clinical application.

## 4. Discussion

### 4.1. Relationship Between Upper Extremity Function and Independence in Activities of Daily Living

The present exploratory cross-sectional pilot study designed to generate hypotheses rather than test them identified a strong negative association between total PRTEE scores and total SCIM III scores (r_s_ = −0.76). Given the limited sample size, these findings should be interpreted as hypothesis-generating rather than confirmatory and do not support predictive inferences. Although univariate regression suggested a statistical relationship, regression estimates derived from such a small dataset may be unstable and are therefore presented for exploratory interpretation only.

The PRTEE primarily evaluates performance aspects dependent on the functional integrity of the elbow, wrist, and hand [21]. Among individuals with tetraplegia, elbow function is particularly critical for independence in ADLs. Improved elbow control enhances reach within living and working environments, reduces reliance on compensatory shoulder external rotation and elbow locking, and supports fine hand movements [31]. Effective grasp and release, combined with appropriate muscle recruitment for tasks requiring fine motor coordination, are essential for functional autonomy [32]. Importantly, U.E. capacity also contributes to postural stabilization during weight shifting, transfer execution, wheelchair propulsion, and protective reactions, thereby linking limb function directly to balance and mobility-related independence.

Because the PRTEE was originally developed for lateral elbow tendinopathy, its application in a neurological population warrants careful consideration. In the absence of widely available patient-reported U.E. tools in Greek, the instrument was selected pragmatically to capture perceived pain and task-related difficulty across activities involving the elbow, wrist, and hand. However, construct validity for individuals with tetraplegia has not been firmly established; therefore, the observed associations should be interpreted cautiously. Several additional measurement limitations merit explicit acknowledgement. First, there is a construct mismatch between tendinopathy-related dysfunction and SCI-related U.E. impairment: the underlying pathological mechanisms differ substantially, and items may not adequately capture the neuromuscular and spastic components of SCI-related dysfunction. Second, the modification of one item (replacing “work” with “phone/computer”) alters the original instrument structure and may affect inter-study comparability. Third, the bilateral administration instruction deviates from the PRTEE’s original unilateral design, which may introduce systematic bias. Collectively, these adaptations raise questions about dimensional stability and measurement equivalence in this population, and the findings should be interpreted accordingly.

These findings are partially consistent with prior research. Lili et al. [18] investigated 25 individuals with cervical or thoracic SCI and identified moderate correlations (r_s_ ≥ 0.6) between kinematic measures of movement smoothness and duration during a drinking task and the SCIM III self-care subscale. Among measures of grip strength and U.E. muscle function, these kinematic parameters showed the strongest associations with self-care. Differences between performance-based metrics and the present patient-reported approach may partly explain variations in correlation patterns. In contrast, in the present study, the total PRTEE score correlated more strongly with the SCIM III respiration and sphincter management subscale (r_s_ = −0.95) and the mobility subscale (r_s_ = −0.74) than with the self-care subscale (r_s_ = −0.69). Lili et al., however, did not report significant correlations with the first two subscales, highlighting possible differences in study populations or methodologies. The particularly strong association observed between PRTEE scores and the SCIM III respiration and sphincter management subscale warrants mechanistic consideration. In individuals with cervical SCI, respiratory function is compromised not only by direct impairment of respiratory musculature (diaphragm, intercostals, abdominals) but also by the reduced capacity for thoracic stabilization that normally supports efficient breathing mechanics [33]. Upper extremity movements generate substantial demands on the trunk as a stabilizing base; in individuals with tetraplegia, the interdependence between arm function and trunk co-activation means that impaired U.E. capacity may reduce the postural support available for respiratory mechanics [9]. Specifically, forced expiration, cough efficiency, and airway clearance all depend on abdominal and thoracic muscle recruitment that is facilitated by a stable U.E. and trunk complex [33]. The findings of a systematic review support that abdominal training have a positive impact in reinforce expiratory flow and therefore improving expiratory muscle recruitment and decreasing airway resistance [34]. Similarly, evidence from a study noted an association between trunk stability and respiratory control in paralympic athletes [35]. Additionally, bladder and bowel management tasks included in the SCIM III respiration/sphincter domain frequently require effortful straining maneuvers that depend on intra-abdominal pressure generation, which is in turn supported by U.E. weight-bearing and trunk stabilization [3,9]. These considerations suggest that the unexpectedly strong correlation between PRTEE and the SCIM III respiration/sphincter domain may reflect shared neuromuscular substrate and functional interdependence between upper limb loading, trunk stabilization, and visceral pressure management in this population, rather than a direct mechanical link between elbow/wrist function and respiration per se.

Similarly, in a study of 50 participants with SCI up to the T6 neurological level, the CUE-T test showed higher correlations with the SCIM III self-care subscale (r_s_ = 0.70) than with the mobility subscale (r_s_ = 0.55) [11]. Consistent with the present findings, Rudhe and van Hedel, in 29 individuals with tetraplegia due to SCI, reported strong correlations between GRASSP hand ability tests and both total SCIM III score (r_s_ = 0.76) and the self-care subscale (r_s_ = 0.80), but lower correlations with the respiration and sphincter management (r_s_ = 0.65) and mobility subscales (r_s_ = 0.72) [17].

Regarding the interpretation of U.E. function in relation to ADL independence, some studies have reported divergent results. Rudhe and van Hedel [16] concluded that hand ability tests were not superior to muscle strength tests in estimating SCIM III self-care scores, suggesting that a combination of five muscle strength tests and one hand ability test provides the most accurate explanatory model. Similarly, Velstra et al. [36], in 61 individuals with acute cervical SCI, found that only muscle strength tests significantly explained variance in independence, whereas hand ability tests did not.

Overall, the strength of associations observed across studies appears heterogeneous, likely reflecting differences in injury severity, assessment methods, and analytic strategies. The present results should therefore be viewed as preliminary evidence supporting a potential relationship between perceived U.E. function and multidimensional independence, particularly in tasks requiring trunk control, transfers, and mobility.

### 4.2. Relationship Between Upper Extremity Pain and Independence in Activities of Daily Living

The PRTEE pain subscale showed weak correlations with the self-care subscale (r_s_ = −0.29) and total SCIM III score (r_s_ = −0.19), suggesting that perceived task difficulty may be more closely aligned with functional independence than pain intensity within this small cohort.

Nonetheless, prior studies indicate that pain directly affects functional independence among individuals with SCI, significantly impacting daily activities and participation [37,38,39]. Over time, individuals with SCI often develop musculoskeletal pain and overuse syndromes in addition to neuropathic pain, since many functional tasks require repetitive U.E. movements, particularly involving the shoulder complex [40,41].

The limited association observed here should not be interpreted as evidence that pain is clinically unimportant. Rather, variability in pain experiences, compensatory strategies, and adaptive behaviors may allow individuals to complete essential ADLs despite discomfort. Given the exploratory design and small sample, these findings warrant confirmation in larger cohorts using multidimensional pain assessment.

### 4.3. Relationship of PRTEE Items with Independence in Activities of Daily Living

Analysis of individual PRTEE function items revealed that “wring out a washcloth/wet towel” (r_s_ = −0.86 for self-care; r_s_ = −0.83 for total SCIM III) and “pull up pants” (r_s_ = −0.85; r_s_ = −0.86, respectively) demonstrated the strongest correlations with both the SCIM III self-care subscale and total score. These activities require coordinated bilateral U.E. engagement and trunk stabilization, reinforcing the biomechanical linkage between arm function, postural control, and independence in transfers and dressing.

These findings also underscore the critical role of wrist mechanics. Individuals with limited finger mobility but approximately 90° of wrist extension can compensate via the tenodesis grasp mechanism, achieving passive digital flexion [9,32]. However, it should be acknowledged that some PRTEE items conceptually overlap with SCIM III tasks (e.g., dressing), which may have inflated correlation estimates and should be considered when interpreting the magnitude of associations.

Overall, these results highlight the importance of bilateral coordination and support incorporating targeted bimanual activity training into rehabilitation programs for individuals with tetraplegia to enhance functional independence. Future research should confirm whether patient-reported task difficulty corresponds with objective performance measures.

### 4.4. Relationship of Injury Related Characteristics with Independence in Activities of Daily Living

Among injury-related factors, only ASIA score demonstrated statistically significant correlations with SCIM III scores. Specifically, ASIA scores showed good correlation with SCIM III respiration/bladder subscale (r_s_ = 0.60) and very good correlations with self-care (r_s_ = 0.86), mobility (r_s_ = 0.85), and total SCIM III score (r_s_ = 0.84). All participants were classified as either C or D on the ASIA scale. suggesting that distinctions between these grades meaningfully impact independence among individuals with tetraplegia.

The ASIA classification reflects injury severity and the extent of motor and sensory function below the neurological level. Individuals classified as ASIA D retain a greater number of muscles moving against gravity (muscle strength ≥ 3), facilitating participation in ADLs such as feeding, dressing, grooming, and bathing, which require active shoulder external rotation, elbow extension, and thumb/finger flexion and abduction [9,32]. These results align with previous studies showing higher independence among individuals with milder injury severity (ASIA D) compared with more severe injuries (ASIA A–C) [42,43]. Similarly, regression analyses in 121 individuals with SCI indicate that ~50% of ASIA D participants can expect full recovery of functional independence [44].

Neurological level of injury showed generally insignificant correlations with SCIM III scores (r_s_ < 0.25), except for a small correlation with respiration/bladder (r_s_ = 0.46). Although literature suggests that more caudal lesions permit greater autonomy [32], some studies align with our findings, reporting small or non-significant correlations [45,46], while others identify neurological level as a significant predictor of ADL independence [17,28,42,43,47]. The lack of significant associations here may reflect the small sample size, limited variability in neurological levels, or the influence of rehabilitation quality.

Time since injury did not significantly correlate with SCIM III scores, suggesting that years living with SCI did not directly influence independence in this sample. Similar results have been reported for mixed tetraplegic and paraplegic populations [46,48]. However, other studies have reported predictive effects of injury duration on functional decline [28,42,49], with some suggesting increases in independence during the first 10 years post-injury, followed by declines between 10 and 20 years [50]. while others reported long-term adaptation leading to greater independence [51]. These findings along with the conflicting evidence in the literature indicate that longitudinal designs are needed to clarify how chronic adaptation, aging, and secondary complications interact to influence long-term autonomy.

### 4.5. Participant Characteristics and Their Association with Functional Independence

Participants’ age (median = 50 years, IQR = 43–55) demonstrated positive Spearman correlations with total SCIM III score (r_s_ = 0.71) as well as with its subscales: self-care (r_s_ = 0.65), respiration/bladder management (r_s_ = 0.65), and mobility (r_s_ = 0.69). Univariate linear regression also showed an association between age and overall SCIM III score. However, this finding should be interpreted with extreme caution: it is very likely a statistical artifact arising from the small and non-representative sample, and it is inconsistent with the broader literature. This association is highly unstable and should not be generalised. Sampling bias (convenience sampling from specialist centres) and survivor bias (older participants who remain independent may be over-represented in outpatient settings) represent plausible explanations for this counterintuitive direction of effect. In contrast, existing literature generally reports that age-related factors—such as decreased muscle strength [28], secondary complications, and comorbidities (e.g., osteoarthritis, cardiovascular disease, type II diabetes) tend to reduce functional independence in individuals with SCI [52,53]. However, older individuals may also possess greater experience and adaptive strategies, which can offset some age-related declines. Evidence on the influence of age is mixed: while several studies identify age as a predictor of reduced SCIM III performance in both tetraplegic and paraplegic populations [28,47,49,51,54,55], others find no significant effect [46,48]. For example, Majamäki et al. [42], in a cross-sectional study of 1772 Finnish individuals with SCI, observed that participants aged ≥ 60 years had the lowest likelihood of independence, whereas those aged 31–45 years had lower subscale scores compared with the 45–60 age group.

Body mass showed a fair correlation with total SCIM III (rs = 0.34), self-care (rs = 0.38), and mobility (rs = 0.33) scores, but was not a statistically significant predictor of independence in ADLs. Literature indicates that BMI can influence functional independence, particularly when obesity is combined with muscle atrophy [28,46]. Tanaka et al. [56], in a study of 154 individuals with tetraplegia, reported that overweight and obese participants had higher SCIM scores in multivariable regression, suggesting that muscle mass preservation may be more relevant than body weight alone.

Height and physical activity level were negligibly correlated with functional independence, except for a fair correlation between physical activity and the respiration/bladder subscale (rs = 0.39). Neto et al. [28] similarly found in 54 individuals with paraplegia that these variables were not significant predictors of independence.

Finally, the type of mobility aid demonstrated good correlations with both the mobility subscale (rs = 0.66) and total SCIM III score (rs = 0.65). Participants using powered wheelchairs generally exhibited lower levels of functional independence. Similarly, Zhao et al. [47], in a study of 228 children with SCI, observed that the presence or absence of a mobility aid correlated with functional independence; however, multivariable regression indicated that it was not an independent predictor. This association likely reflects the underlying severity of SCI (ASIA classification), which determines the type of mobility aid required.

### 4.6. Limitations

This study has several limitations that should be acknowledged. First, the sample size was smaller than the threshold required for adequate statistical power, which may reduce estimate precision and limit generalizability to the broader SCI population. The study is therefore markedly underpowered relative to the a priori sample size calculation, increasing the risk of both Type II error and unstable effect estimates. Readers are strongly cautioned against over-interpreting any specific correlation coefficients or regression estimates reported here; all quantitative findings must be treated as preliminary and hypothesis-generating, requiring replication in larger, adequately powered, and more representative samples before any clinical conclusions can be drawn.

Additionally, the sample included only male participants; although this likely reflects the available clinical population, it may further constrain external validity. The convenience sampling approach used in this study introduces a further risk of selection bias: participants who responded and completed all assessments may differ systematically from non-responders or from the broader population of individuals with motor-incomplete tetraplegia. The small and homogeneous sample (all male, single-country, community-dwelling) substantially limits the generalisability of findings to women, to individuals in acute or inpatient settings, and to SCI populations in other healthcare contexts.

Second, participant heterogeneity and the cross-sectional design preclude causal inference and permit only the identification of associations. Third, the PRTEE was adapted for this neurological cohort, including modification of the work-related item and mean imputation for non-applicable responses in accordance with scoring recommendations [34]. While pragmatically necessary, these procedures may have influenced measurement equivalence and should be considered when interpreting the findings.

Fourth, reliance on self-reported instruments may introduce recall and perception bias; future investigations should incorporate performance-based assessments to enhance construct validity. In the absence of a validated Greek version of the SCIM IV, the SCIM III was employed. Although widely accepted in SCI research, it may not fully reflect the most recent refinements of the instrument.

Fifth, the study was underpowered relative to the a priori sample size calculation (*N* = 11 vs. required *N* = 29), which substantially increases the risk of both Type II error and unstable effect estimates; regression coefficients in particular should be treated as illustrative only.

Sixth, multiple bivariate correlations were examined without adjustment for multiplicity, increasing the risk of Type I error; accordingly, all correlation findings should be interpreted as exploratory and hypothesis-generating.

Seventh, conceptual overlap between certain PRTEE items and SCIM III tasks (e.g., dressing) may have inflated correlation estimates. Although sensitivity analyses excluding overlapping items yielded directionally consistent associations, residual inflation cannot be entirely excluded.

Eighth, the PRTEE was not originally developed for neurological populations. Its adaptation in the present study—including item modification and mean imputation—raises concerns regarding construct validity, dimensional stability, and measurement equivalence that warrant further psychometric evaluation.

Finally, consistent with Babyak’s (2004) [30] discussion of overfitting, model complexity was deliberately constrained to remain proportionate to the available sample size. The revised analytic strategy was therefore intentionally conservative to mitigate the risk of spurious inference.

## 5. Conclusions

In this exploratory cross-sectional pilot study designed to generate hypotheses rather than test them, self-reported U.E. function showed negative associations with independence in activities of daily living among individuals with chronic motor-incomplete tetraplegia. These associations should be interpreted as preliminary, hypothesis-generating observations only. Given the small sample, absence of multiple comparison correction, potential conceptual overlap between instruments, and the exploratory application of the PRTEE in a neurological population, no confirmatory or predictive inferences can be drawn. The observed associations support a potential functional linkage between perceived arm capacity and ADL independence but require replication in adequately powered studies using validated neurological outcome measures.

The findings underscore the clinical relevance of comprehensive U.E. assessment when evaluating functional status; however, the small sample and methodological constraints necessitate cautious interpretation. Future research with adequately powered samples, validated neurological outcome measures, and performance-based testing is essential to confirm these observations and guide rehabilitation planning.

## Figures and Tables

**Table 1:** Participants’ Demographic Characteristics (*N* = 11).

Demographic Characteristic	Median (IQR)/*N* (%)
Gender	
Male	11 (100)
Female	0
Age (years)	50 (43–55)
Body Mass (kg)	82 (70–98)
Height (cm)	180 (172–186)
Walking Aid	
Powered Wheelchair	5 (45.5)
Manual Wheelchair	3 (27.3)
Walker	1 (9.1)
Crutches	0
None	2 (18.2)
Physical Activity Level	
Sedentary (0 h)	2 (18.2)
Moderately Active (1–3 h)	3 (27.3)
Active (3–6 h)	2 (18.2)
Very Active/Athlete (>6 h)	4 (36.4)

Data are presented as median (IQR, Q1–Q3) or as absolute frequencies and percentages (%) depending on variable type.

**Table 2:** Participants’ Injury-Related Characteristics (*N* = 11).

Injury Related Characteristic	Median (IQR)/*N* (%)
Neurological Level of Injury	
C4	1 (9.1)
4–C5	1 (9.1)
C5	1 (9.1)
C5–C6	2 (18.2)
C6	3 (27.3)
T1	3 (27.3)
ASIA Classification	
C	7 (63.6)
D	4 (36.4)
Time since Injury (years)	12 (7–16)
SCI Etiology	
Traffic Accident	5 (45.5)
Myelopathy	4 (36.4)
Athletic Injury	2 (18.2)

Data are presented as median (IQR, Q1–Q3) or as absolute frequencies and percentages (%) depending on variable type.

**Table 3:** Participants’ PRTEE Score (*N* = 11).

Section/Item Evaluation	Median (IQR)
U.E Pain	19 (10–24)
U.E Function	25 (20–40)
Turn a Doorknob/Key	5 (3–10)
Carry a Grocery Bag/Briefcase by the Handle	4 (0–10)
Lift a Full Cup/Glass to your Mouth	6 (1–9)
Open a Jar	6 (4–10)
Pull up Pants	10 (2–10)
Wring out a Washcloth/Wet Towel	7 (1–10)
Personal Activities	9 (7–10)
Household Work	10 (7–10)
Work (phone—computer)	1 (0–5)
Recreational/Sporting Activities	4 (2–7)
Total Score	46 (41–59.5)

Data are presented as median (IQR, Q1–Q3).

**Table 4:** Participants’ SCIM III Score (*N* = 11).

Section/Item	Median (IQR)
Self-Care	10 (1–16)
Feeding	2 (1–3)
Bathing—Upper Body	2 (0–2)
Bathing—Lower Body	0 (0–2)
Dressing—Upper Body	2 (0–3)
Dressing—Lower Body	0 (0–3)
Grooming	1 (0–3)
Respiration and Sphincter Management	26 (21–35)
Mobility (Room and Toilet)	17 (3–27)
Total Score	54 (25–74)

Data are presented as median (IQR, Q1–Q3).

**Table 5:** Explanatory Variable’s Correlation with SCIM III Scores.

Explanatory Variable	Self-Care Score		Respiration/Sphincter Score		Mobility Score		Total Score	
	rs	*p*-Value	rs	*p*-Value	rs	*p*-Value	rs	*p*-Value
PRTEE Pain Score	−0.29	0.39	−0.32	0.33	−0.22	0.53	−0.19	0.57
PRTEE Function Score	−0.94 **	<0.01	−0.83 **	<0.01	−0.92 **	<0.01	−0.93 **	<0.01
PRTEE Total Score	−0.69 *	0.02	−0.95 **	<0.01	−0.74 *	0.01	−0.76 **	0.01
Age. years	0.65 *	0.03	0.65 *	0.03	0.69 *	0.02	0.71 *	0.02
Body Mass. kg	0.38	0.25	0.2	0.55	0.33	0.32	0.34	0.31
Height. cm	−0.04	0.9	−0.12	0.72	−0.15	0.67	−0.16	0.65
Walking Aid	0.58	0.06	0.18	0.61	0.66 *	0.03	0.65 *	0.03
Physical Activity Level	0.01	0.99	0.39	0.24	0.12	0.73	0.12	0.73
Neurological Level of Injury	0.08	0.82	0.46	0.16	0.02	0.95	0.08	0.83
ASIA Classification	0.86 **	<0.01	0.6 *	0.05	0.85 **	<0.01	0.84 **	<0.01
Time since Injury. years	0.2	0.55	0.03	0.94	0.11	0.75	0.12	0.72
SCI Etiology	0.29	0.4	0.1	0.76	0.4	0.23	0.37	0.27

Correlation rs = 0.6–0.75 *, Correlation rs > 0.75 **.

**Table 6:** Correlation of PRTEE Specific Activities with SCIM III Self-care and Total Score.

PRTEE Function Item	Self-Care Score		Total Score	
	rs	*p*-Value	rs	*p*-Value
Turn a Doorknob/Key	−0.75 *	0.01	−0.69 *	0.02
Carry a Grocery Bag/Briefcase by the Handle	−0.79 **	<0.01	−0.59	0.06
Lift a Full Cup/Glass to your Mouth	−0.8 **	<0.01	−0.7 *	0.02
Open a Jar	−0.67 *	0.02	−0.75 *	0.01
Pull up Pants	−0.85 **	<0.01	−0.86 **	<0.01
Wring out a Washcloth/Wet Towel	−0.86 **	<0.01	−0.83 **	<0.01

Correlation rs = 0.6–0.75 *, Correlation rs > 0.75 **.

## Data Availability

The data presented in this study are available on reasonable request from the corresponding author. The data are not publicly available due to privacy and ethical restrictions, as they contain sensitive information from individuals with spinal cord injury and are protected in accordance with institutional ethics approval and data protection regulations.

## Abbreviations

The following abbreviations are used in this manuscript:

SCI	Spinal Cord Injury
U.E	Upper Extremity
ADL	Activities of Daily Living
PRTEE	Patient Rated Tennis Elbow Evaluation
SCIM III	Spinal Cord Independence Measure
ASIA	American Spinal Injury Association
IQR	Interquartile Range
rs	Spearman’s rank correlation coefficient
